# Supplementary material for: Local ancestry inference identifies robust evidence of selection in Neolithic Europe
Source: Mol Biol Evol. 2026 Sep 17;43(9):msag236. doi: 10.1093/molbev/msag236 (PMC13614254; doi:10.1093/molbev/msag236)
Supplement: Supplementary file 1 [file msag236_supplementary_data.zip › Mies_LAI_supp_revision_GM.pdf]

Supplemental Information

Local ancestry inference identifies robust evidence of selection in Neolithic Europe

Georgia Mies and Iain Mathieson

Supplementary Figures

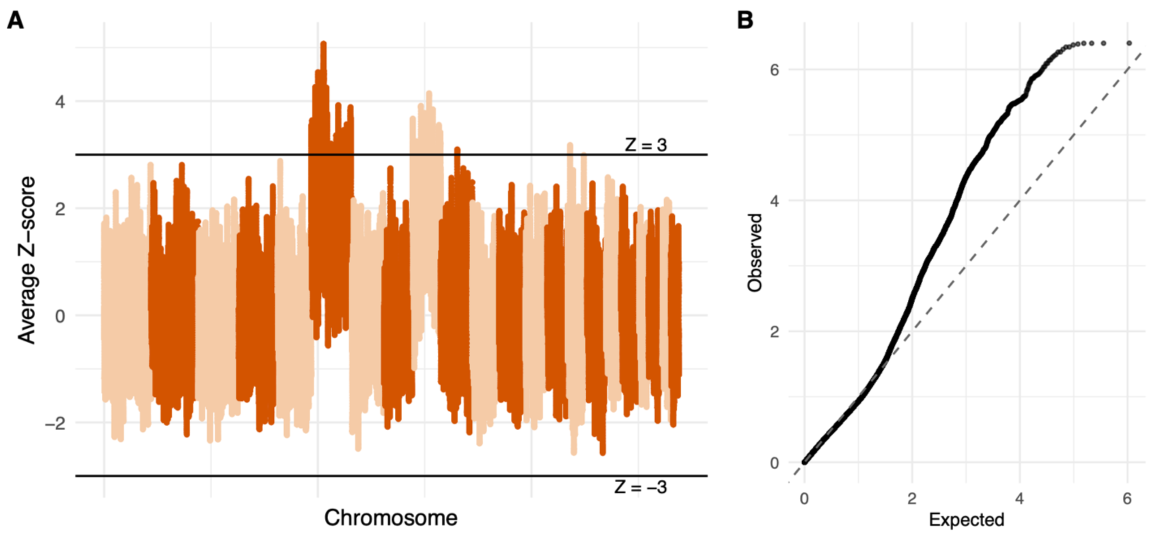

Figure S1. AncestralPaths Z-scores for discovery dataset (N = 176). A) Manhattan plot showing elevated hunter-gatherer ancestry on chromosomes 6 and 9 prior to per-chromosome normalization to the genome-wide average. **B)** QQ plot of Z-scores.

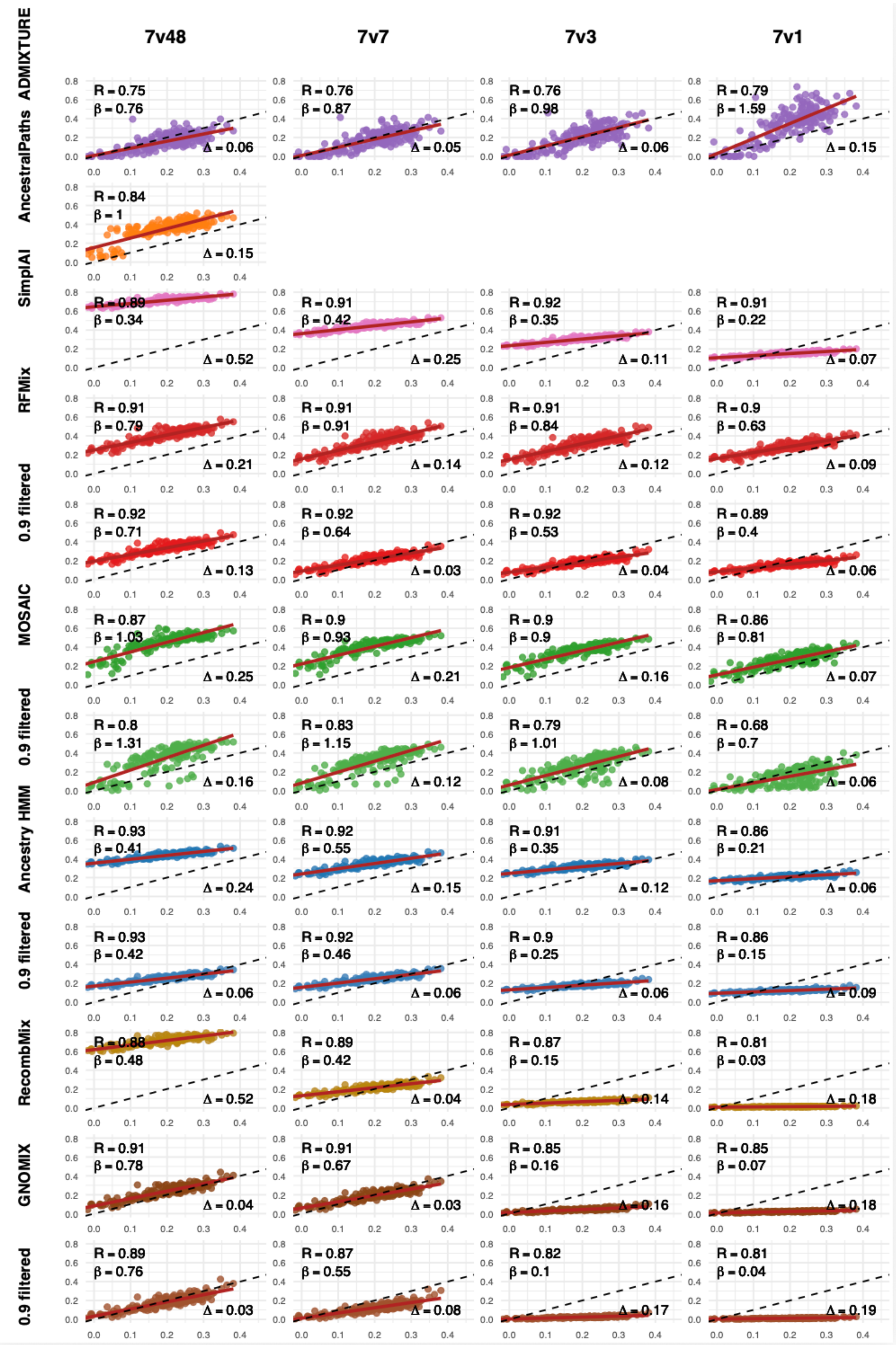
7v48
7v7
7v3
7v1
ADMIXTURE
AncestralPaths
SimplAI
RFMix
0.9 filtered
MOSAIC
0.9 filtered
Ancestry HMM
0.9 filtered
RecombMix
GNOMIX
0.9 filtered
R = 0.75 β = 0.76 Δ = 0.06
R = 0.76 β = 0.87 Δ = 0.05
R = 0.76 β = 0.98 Δ = 0.06
R = 0.79 β = 1.59 Δ = 0.15
R = 0.84 β = 1 Δ = 0.15
R = 0.89 β = 0.34 Δ = 0.52
R = 0.91 β = 0.42 Δ = 0.25
R = 0.92 β = 0.35 Δ = 0.11
R = 0.91 β = 0.22 Δ = 0.07
R = 0.91 β = 0.79 Δ = 0.21
R = 0.91 β = 0.91 Δ = 0.14
R = 0.91 β = 0.84 Δ = 0.12
R = 0.9 β = 0.63 Δ = 0.09
R = 0.92 β = 0.71 Δ = 0.13
R = 0.92 β = 0.64 Δ = 0.03
R = 0.92 β = 0.53 Δ = 0.04
R = 0.89 β = 0.4 Δ = 0.06
R = 0.87 β = 1.03 Δ = 0.25
R = 0.9 β = 0.93 Δ = 0.21
R = 0.9 β = 0.9 Δ = 0.16
R = 0.86 β = 0.81 Δ = 0.07
R = 0.8 β = 1.31 Δ = 0.16
R = 0.83 β = 1.15 Δ = 0.12
R = 0.79 β = 1.01 Δ = 0.08
R = 0.68 β = 0.7 Δ = 0.06
R = 0.93 β = 0.41 Δ = 0.24
R = 0.92 β = 0.55 Δ = 0.15
R = 0.91 β = 0.35 Δ = 0.12
R = 0.86 β = 0.21 Δ = 0.06
R = 0.93 β = 0.42 Δ = 0.06
R = 0.92 β = 0.46 Δ = 0.06
R = 0.9 β = 0.25 Δ = 0.06
R = 0.86 β = 0.15 Δ = 0.09
R = 0.88 β = 0.48 Δ = 0.52
R = 0.89 β = 0.42 Δ = 0.04
R = 0.87 β = 0.15 Δ = 0.14
R = 0.81 β = 0.03 Δ = 0.18
R = 0.91 β = 0.78 Δ = 0.04
R = 0.91 β = 0.67 Δ = 0.03
R = 0.85 β = 0.16 Δ = 0.16
R = 0.85 β = 0.07 Δ = 0.18
R = 0.89 β = 0.76 Δ = 0.03
R = 0.87 β = 0.55 Δ = 0.08
R = 0.82 β = 0.1 Δ = 0.17
R = 0.81 β = 0.04 Δ = 0.19

Figure S2. Full genome ancestry estimated by method for discovery dataset (N = 176). Correlations between global ancestry proportions estimated by ADMIXTURE and LAI methods vs qpAdm. Each point represents one of the 176 admixed Neolithic individuals. R denotes the Pearson correlation between the method and qpAdm individual estimates. β indicates the slope of the regression line between the method and qpAdm estimates. Δ represents the average absolute difference in global ancestry between the LAI method and qpAdm. The dashed line corresponds to y = x for qpAdm estimates, while the red line shows the best-fit linear regression between qpAdm and the LAI method. Sample sizes of sources are listed for each column in the format of 7 farmers v N hunter-gatherers (7vN). Rows are labeled by method and whether calls were filtered by 0.9 posterior probability.

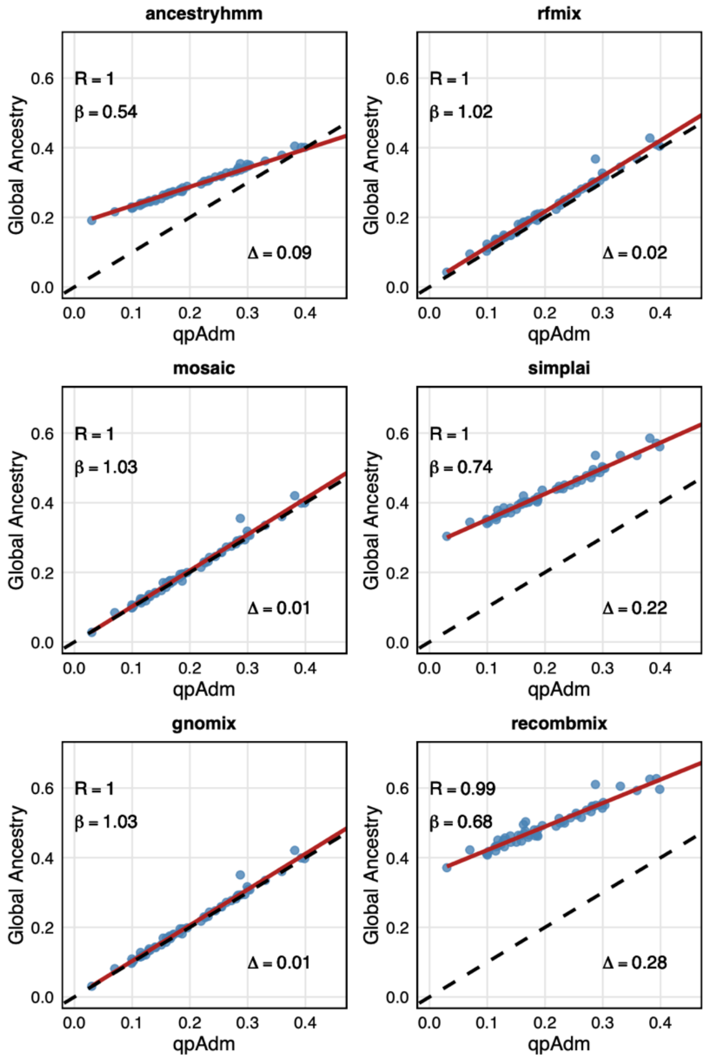

Figure S3. Full genome ancestry estimated by LAI methods in 1000 Genomes Project African Americans. Correlations in European (CEU) global ancestry proportions between LAI method and qpAdm. Each point is one of the 61 admixed African American (ASW) individuals.

R denotes the Pearson correlation between the method and qpAdm individual estimates. β indicates the slope of the regression line between the method and qpAdm estimates. Δ represents the average absolute difference in global ancestry between the LAI method and qpAdm. The dashed line corresponds to y = x for qpAdm estimates, while the red line shows the best-fit linear regression between qpAdm and the LAI method. Sample sizes of sources are 7 European (CEU) and 48 African (YRI) sources for Ancestry HMM, RFMix, Mosaic, Gnomix, Recomb-Mix, and 7v7 for simpLAI.

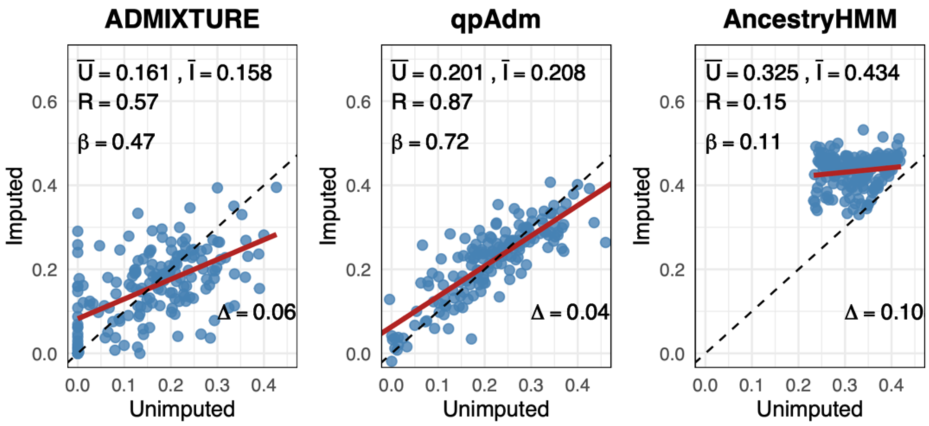

Figure S4. Full genome ancestry estimated for imputed and unimputed data. Figures show correlations in hunter-gatherer global ancestry proportions comparing results from **A)** qpAdm, **B)** ADMIXTURE, and **C)** Ancestry HMM on imputed and unimputed data. Each point is one of the 176 admixed Neolithic individuals. Ū and Ī estimates are mean ancestry estimates for unimputed and imputed data for each method respectively. R denotes the Pearson correlation between the method's individual estimates for unimputed and imputed datatypes. β indicates the slope of the regression line between the method's two datatypes individual estimates. Δ represents the average absolute difference in global ancestry between the method on each datatype. The dashed line corresponds to y = x for unimputed datatype results, while the red line shows the best-fit linear regression between method on unimputed and imputed data. Sample sizes of sources are 7 farmers and 48 hunter-gatherers.

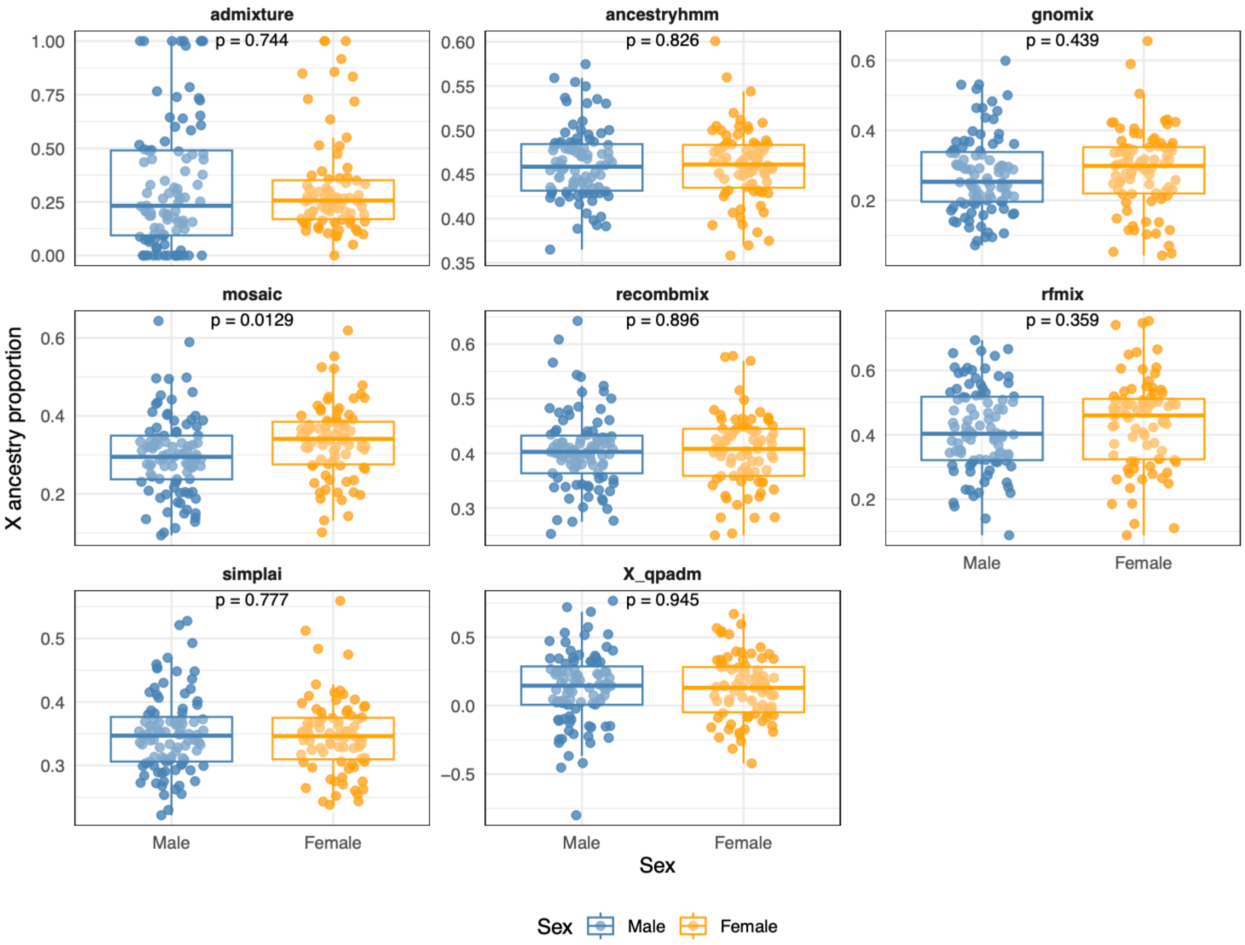

Figure S5. X chromosome discovery dataset (N = 176) hunter-gatherer ancestry by sex. Genome-wide X chromosome ancestry proportions for males and females across LAI methods for discovery dataset. Source sample sizes follow 7vN used in Figure 2. P-values are from two-sided t-tests comparing mean ancestry between sexes. Boxplots show median and interquartile range.

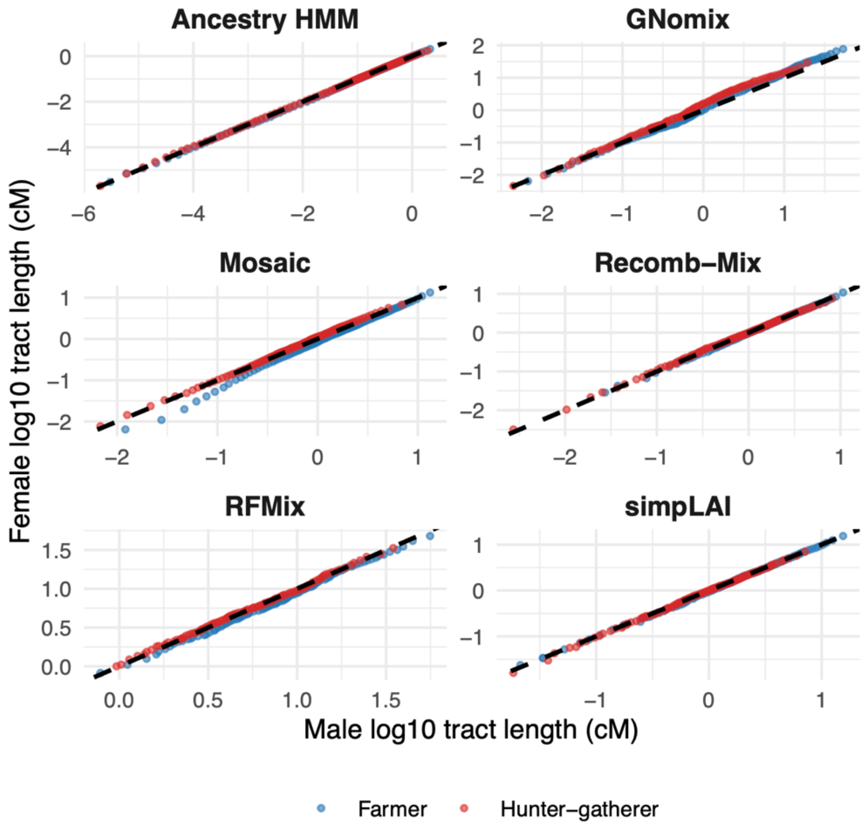

Figure S6. QQ plot of X chromosome tract length distribution for discovery dataset (N = 176). Comparing male and female tract length distributions (in log10 tract length (cM)) for each method, shown separately for farmer and hunter-gatherer ancestries.

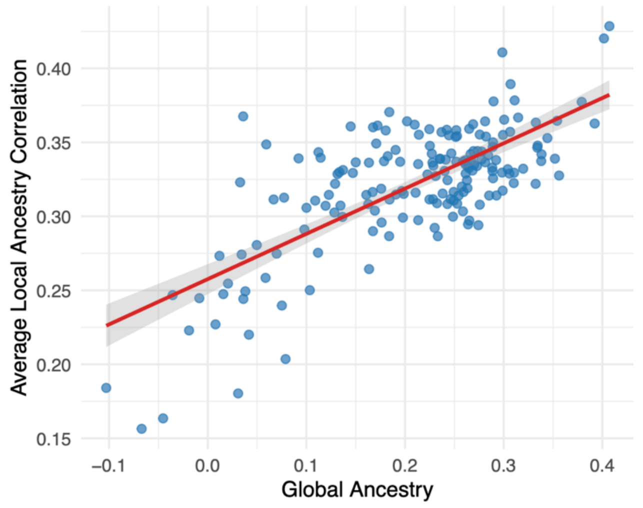

Figure S7. Individual-level average local ancestry correlation across method pairs in discovery dataset (N = 176). Individual-level average pairwise local ancestry correlation across

seven methods, computed in 100 kb bins and plotted against global hunter-gatherer ancestry (qpAdm). Each point represents an individual. The red line indicates the best-fit linear regression. Source sample sizes follow 7vN used in Figure 2.

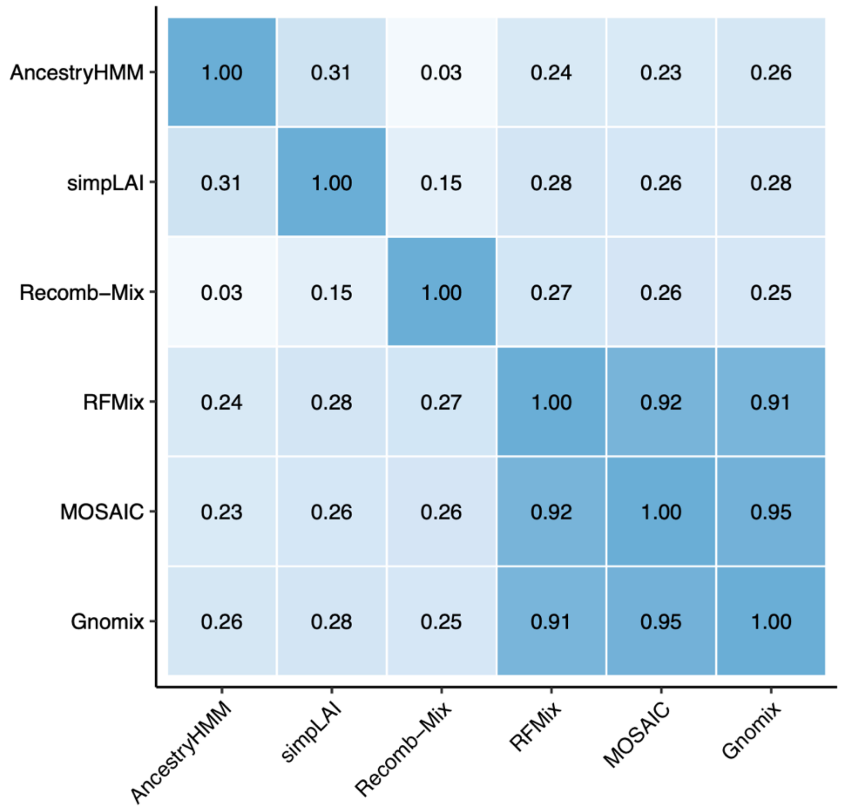

Figure S8. Local ancestry correlations for LAI methods in 1000 Genomes. Correlations in European (CEU) local ancestry proportions between LAI methods in 100kb bins along the genome. Sample sizes of sources are 7 CEU and 48 YRI sources for Ancestry HMM, RFMix, Gnomix, Recomb-Mix and Mosaic, and 7v7 for simpLAI.

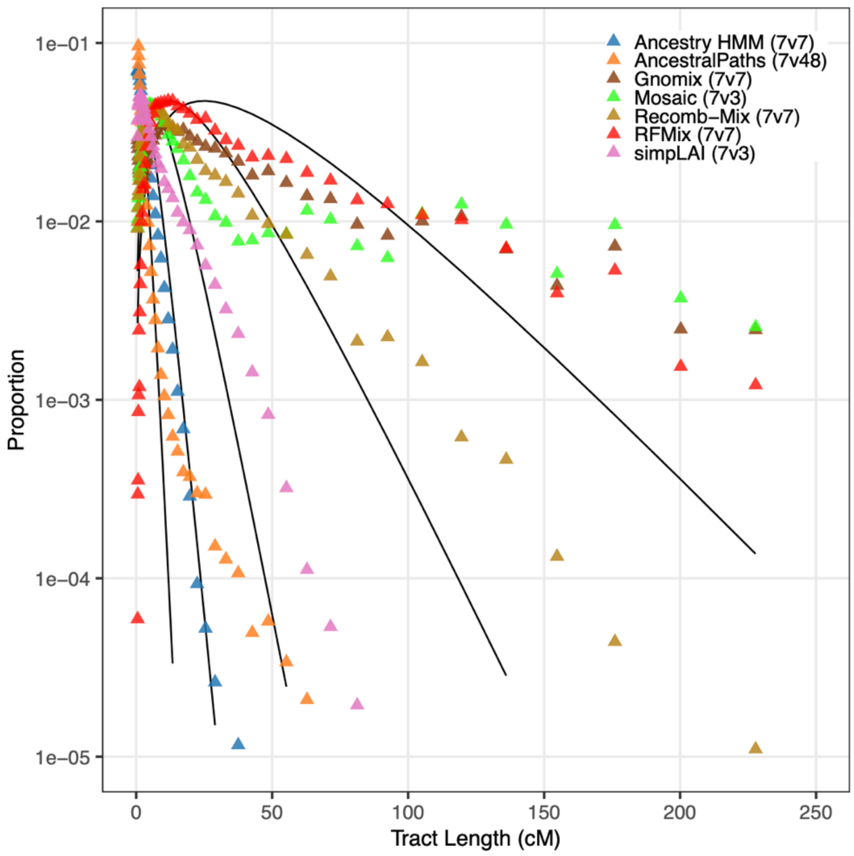

Figure S9. Farmer tract length distributions by local ancestry inference method in discovery dataset (N = 176). Proportion of farmer ancestry tracts by length (in cM) inferred using seven local ancestry inference methods. Empirical tract length distributions are shown as points, and black lines indicate theoretical single-pulse admixture models with admixture times (T) of 200, 100, 50, 20, and 10 generations. The figure provides the corresponding farmer ancestry tract distributions to complement the hunter-gatherer tract length analysis presented in Figure 4 of the main text. Source sample sizes are indicated in parentheses with each method title, representing 7 farmer reference individuals versus the number hunter gatherer sources (7vN).

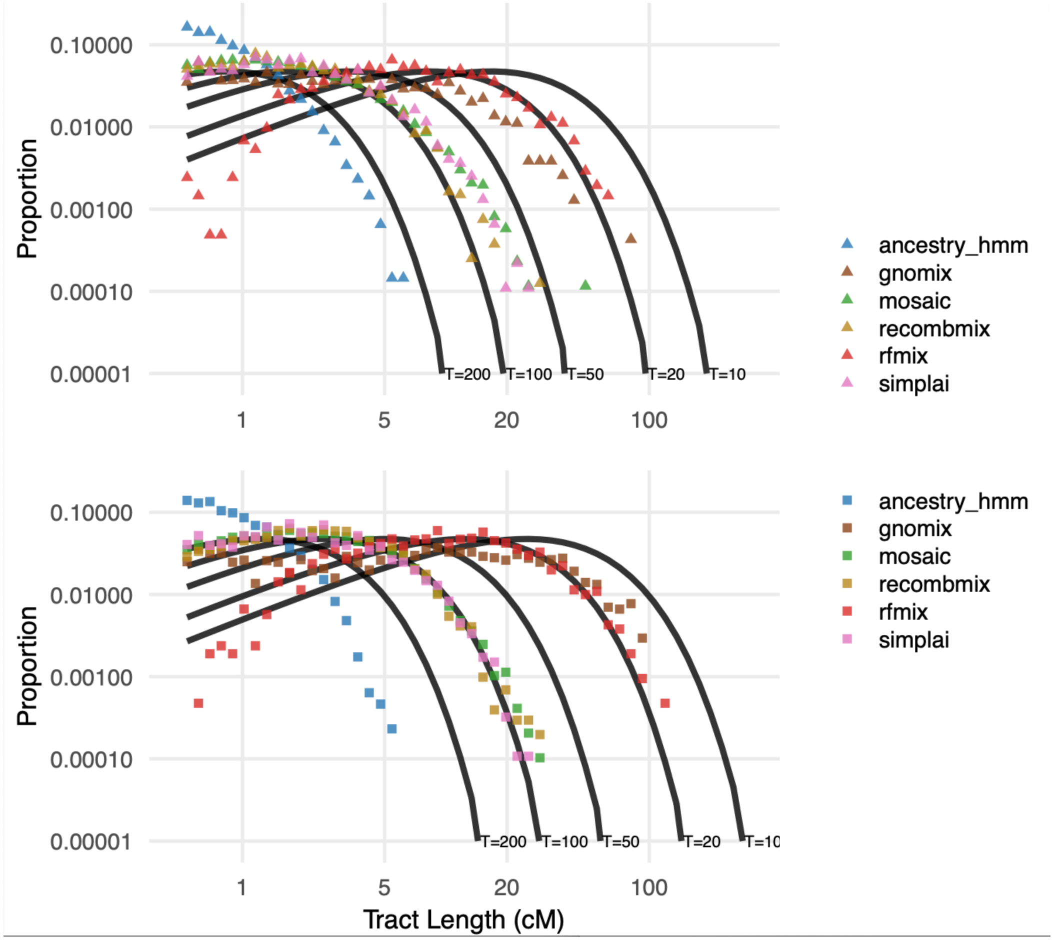

Figure S10. X-chromosome tract length distributions inferred by LAI methods for discovery dataset (N = 176). Distribution of tract lengths (cM) for each method compared to theoretical expectations (T = 200, 100, 50, 20, 10 generations). Top panel shows hunter-gatherer ancestry tract lengths (triangles) and bottom panel shows farmer ancestry tract lengths (squares).

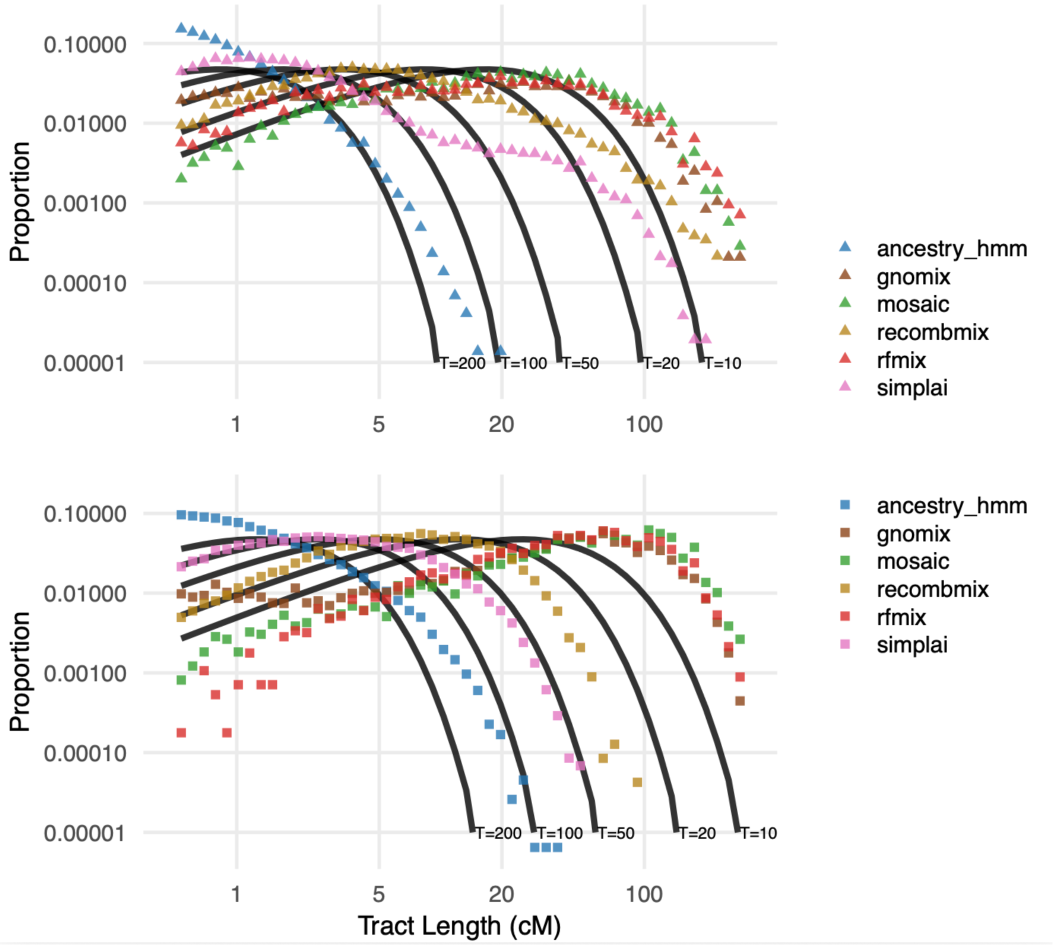

Figure S11. Tract length distributions inferred by LAI methods in 1000 Genomes Project African Americans. Distribution of tract lengths (cM) for each method compared to theoretical expectations (T = 200, 100, 50, 20, 10 generations). Top panel shows CEU ancestry tract lengths (triangles) and bottom panel shows YRI ancestry tract lengths (squares). Sample sizes of sources are 7 CEU and 48 YRI sources for Ancestry HMM, RFMix, Gnomix, Recomb-Mix, and Mosaic, and 7v7 for simpLAI.

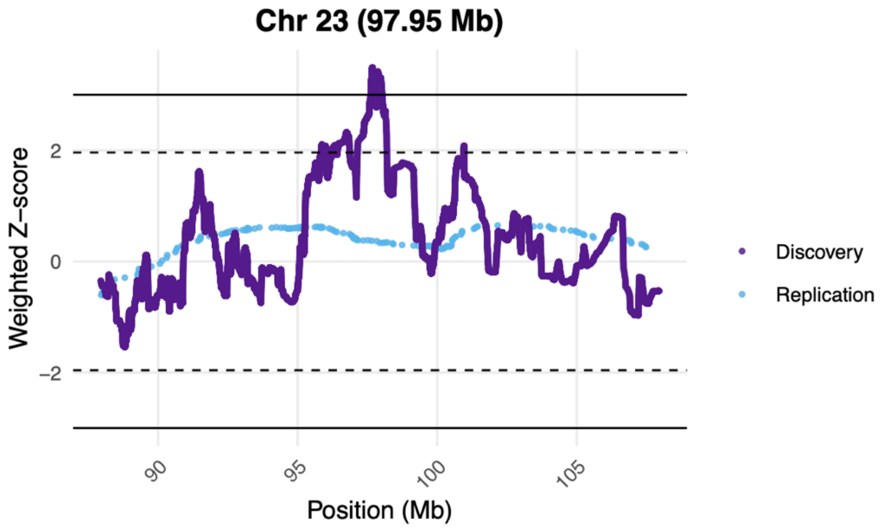

Figure S12. Replication of X chromosome hit. Comparison of combined and replication Z-scores for X chromosome loci exceeding the Z = ±3 discovery threshold. Solid lines indicate the discovery threshold (Z = ±3), and dashed lines indicate the replication threshold (Z = ±1.96). Discovery results (dark purple) are from meta-analysis of the six local ancestry inference methods on X chromosome discovery analyses (N = 176; Mosaic, RFMix, Gnomix, Recomb-Mix, simpLAI), and replication results (light blue) are from unimputed replication dataset 1 analyzed with Ancestry HMM.

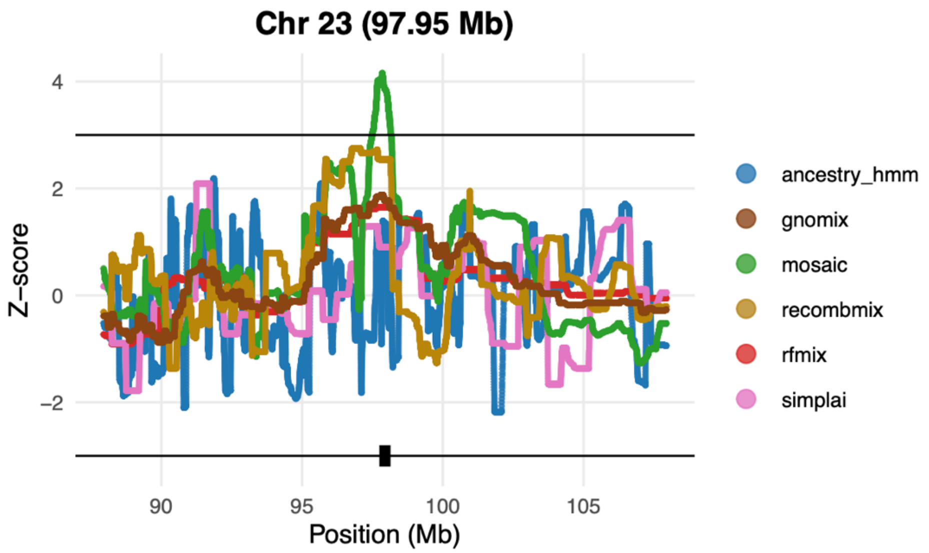

Figure S13. Z-score deviations across methods for X chromosome hit. Z-scores across methods for the discovery dataset (N = 176) X chromosome region (X:97.95 Mb). Horizontal lines indicate Z = ±3 thresholds used in the meta-analysis.

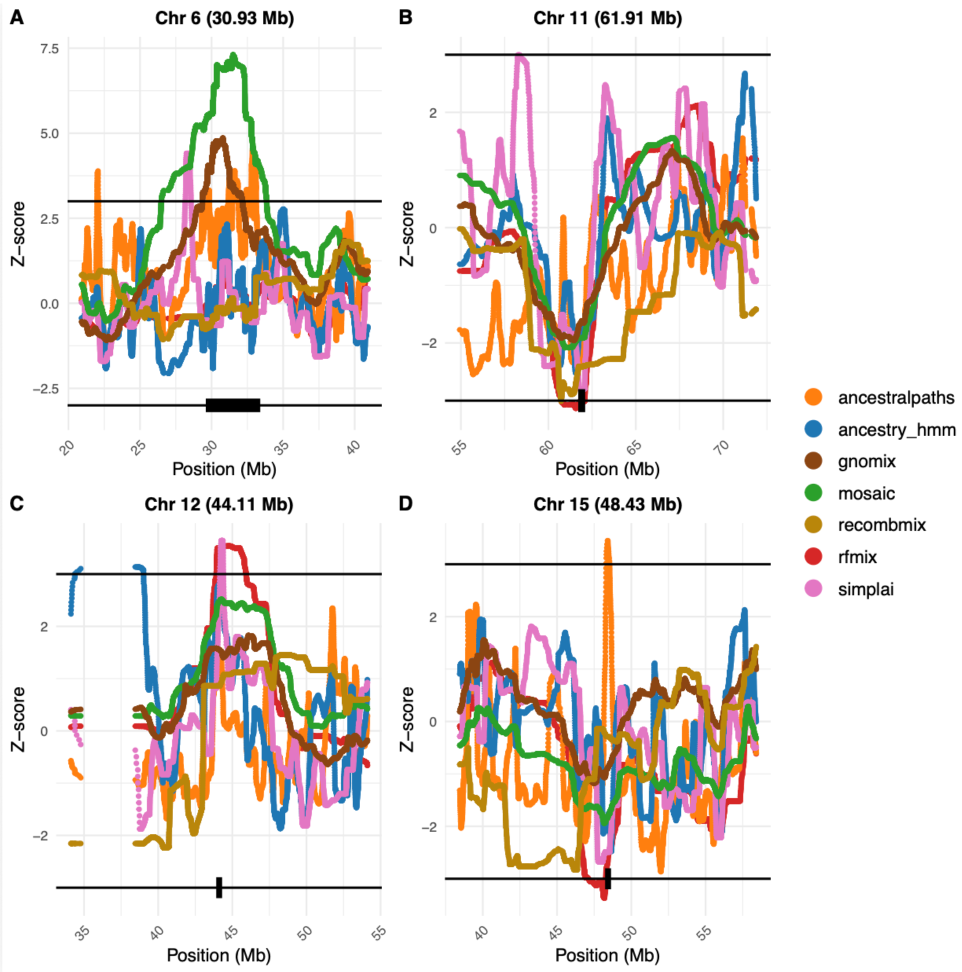

Figure S14. Z-score deviations across methods for replicated loci. Z-scores across methods for the five replicated regions in the discovery dataset. Horizontal lines indicate Z = ±3 thresholds used in the meta-analysis. Methods use 7v48 source panels, except simpLAI (7v7). Putative candidate genes are highlighted with black boxes.

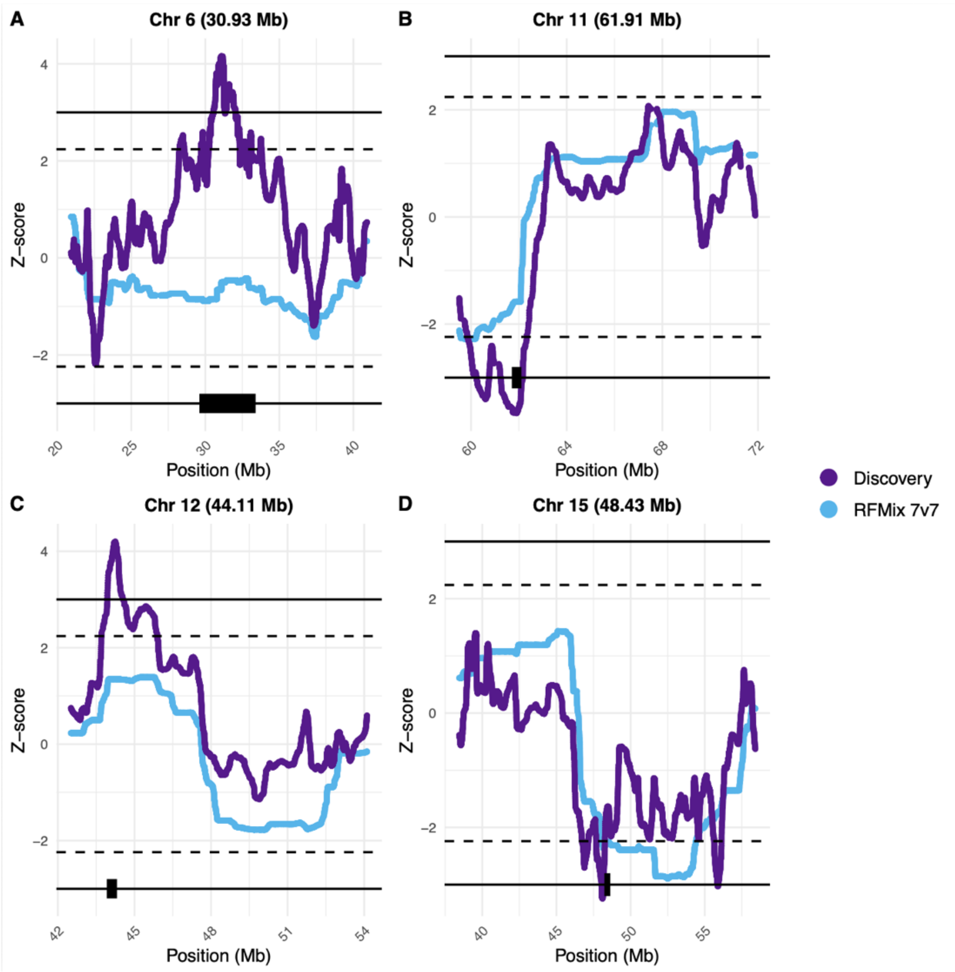

Figure S15. Replication of Z-scores from meta-analysis of discovery dataset in RFMix 7v7 results. Comparison of meta-analysis of seven methods with 7v48 sources Z-scores and RFMix 7v7 Z-scores at the four replicated loci. Dashed lines indicate the discovery threshold (Z = ±3), and solid lines indicate the replication threshold (Z = ±2.64).

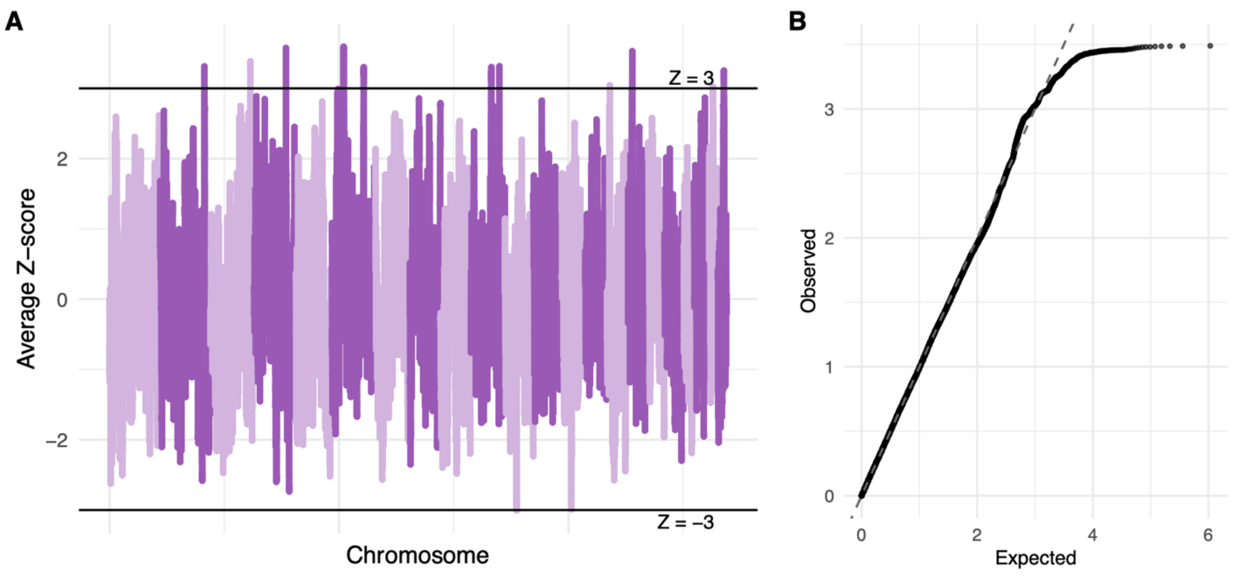

Figure S16. Manhattan and QQ plots for meta-analysis of discovery dataset (N = 176) using 7vN source reference panels. Results for all methods using 7vN source panels. Horizontal dashed lines indicate Z = ±3. Source sample sizes (7vN) correspond to Figure 2.

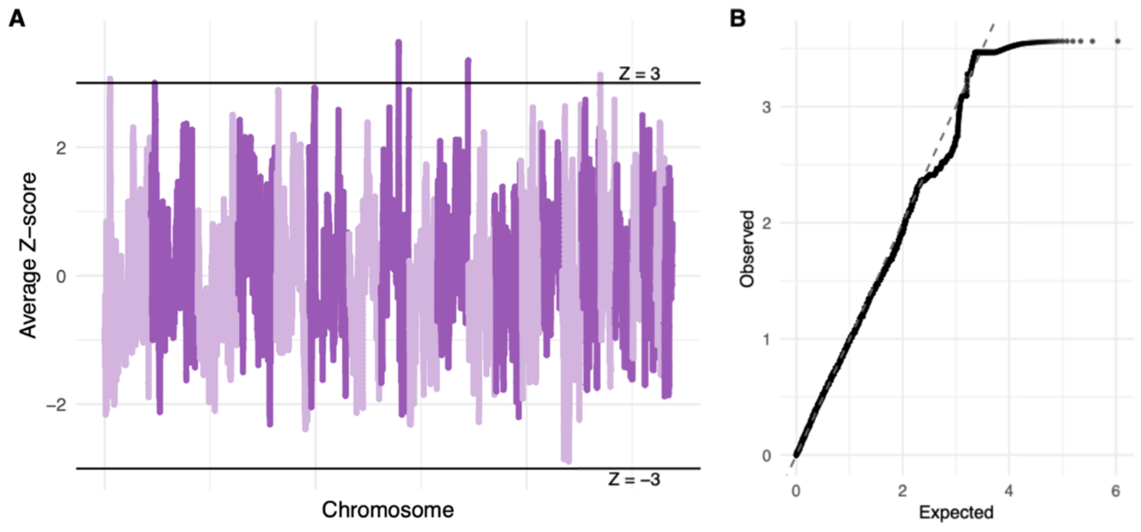

Figure S17. Manhattan and QQ plots for RFMix 7v7. Horizontal dashed lines indicate Z = ±3.
